# Synthesis and field emission properties of different ZnO nanostructure arrays

**DOI:** 10.1186/1556-276X-7-197

**Published:** 2012-03-23

**Authors:** Yaoguo Fang, Kin Mun Wong, Yong Lei

**Affiliations:** 1 Institute of Materials Physics and Center for Nanotechnology, University of Muenster, Wilhelm-Klemm-Str. 10, Muenster 48149, Germany; 2 Institut für Physik & IMN MacroNano® (ZIK), Technische Universität Ilmenau, Prof. Schmidt-Str. 26, Ilmenau 98693, Germany

**Keywords:** ZnO, Nanowires, Structure directing chemicals, Field emission properties

## Abstract

In this article, zinc oxide (ZnO) nanostructures of different shapes were fabricated on silicon substrate. Well-aligned and long ZnO nanowire (NW) arrays, as well as leaf-like ZnO nanostructures (which consist of modulated and single-phase structures), were fabricated by a chemical vapor deposition (CVD) method without the assistance of a catalyst. On the other hand, needle-like ZnO NW arrays were first fabricated with the CVD process followed by chemical etching of the NW arrays. The use of chemical etching provides a low-cost and convenient method of obtaining the needle-like arrays. In addition, the field emission properties of the different ZnO NW arrays were also investigated where some differences in the turn-on field and the field-enhancement factors were observed for the ZnO nanostructures of different lengths and shapes. It was experimentally observed that the leaf-like ZnO nanostructure is most suitable for field emission due to its lowest turn-on and threshold field as well as its high field-enhancement factor among the different synthesized nanostructures.

## Background

One-dimensional (1-D) metallic oxide nanostructures have been extensively studied due to their numerous applications as basic building blocks in a variety of nanodevices. Among those 1-D nanostructures, zinc oxide (ZnO) with a wide bandgap (3.37 eV) and a large exciton binding energy (60 meV) at room temperature has attracted a lot of attention in the last few years. Such 1-D ZnO wire-like arrays have acted as a vital candidate for applications in gas sensor [1], dye-sensitized solar cells [2], UV photodetectors [3,4], nanowire laser [5], light-emitting diodes [6], UV sensors [7], field-effect transistors [8], nanogenerators [9], field emission devices [10], and so on. Among them, the properties of electron field emitters have been extensively investigated using ZnO wire-like arrays because they have negative electron affinity, high mechanical strength, chemical stability, and aspect ratios [11,12]. Hence, different morphologies of the ZnO nanostructures have been synthesized [13] such as wire-like ZnO with high aspect ratios and small tip radius of curvature, which has been prepared in recent years. Lately, 1-D indium (In)-doped ZnO superlattice structures were prepared under the assistance of catalysts or precursors at high temperature. Those structures consisted of alternated In-doped ZnO and In-O layers growing along the c-axis growth direction [14-17]. To date, there are few reports about two-dimensional (2-D) ZnO superlattice structures along the a/b axis growth direction due to Indium doping. Hence, in this work, we report the superlattice phenomenon of In_2_O_3_(ZnO)*_m _*by In doping. Compared with the previous study, the procedure of synthesizing the In-doped ZnO nanostructure reported in this article is simpler, and the use of precursors or catalysts (such as Co_2_O_3_, Sn, Au, and Ce) are not required [14-18]. On the other hand, various methods have been developed for synthesizing the 1-D ZnO nanostructures, such as chemical vapor deposition (CVD) [19], solution-based synthesis [20], hydrothermal methods [21], anodization [22], microwave-assisted aqueous synthesis [23], chemical vapor transport and condensation process [24], rf magnetron sputtering [25], and a modified cabothermal reduction method for preparing large-scale ZnO nanowires [26]. However, it is difficult to prepare vertically long wire-like ZnO NWs using the hydrothermal and microwave methods. In this paper, we report self-assembly of the long ZnO nanowire arrays without the use of catalyst on a silicon (100) substrate by the CVD method where the length of nanowires is about 310 μm after a reaction time of 60 min. Zhou et al. [27] had investigated the field emission properties of tin dioxide (SnO_2_) nanowire arrays, and in this article, the field emission properties of the different synthesized ZnO nanostructures were investigated. Some differences in the field-enhancement factors between the different nanostructures were observed. This information could be useful for the application of the ZnO nanostructures as field emitters.

## Methods

### Experimental procedure

#### Synthesis of the leaf-like indium-doped ZnO nanostructuresss

Leaf-like structures were prepared by a carbothermal reduction process without any catalyst. After grinding, 0.45 g of ZnO (purity, 99.999%), 0.05 g of In_2_O_3 _(purity, > 99%), and 0.5 g of fine graphite powder were dispersed in a quartz boat placed in an alumina tube (length is 150 cm and inner diameter is 58 mm) at the center of the furnace. Silicon substrates (100) were washed by alcohol and acetone in an ultrasonic bath for half an hour, respectively, and were then located downstream from the source (3 to 5 mm) in the quartz boat. Argon (80 sccm) and 2.5 sccm of oxygen were introduced into the chamber, and the system was pumped to a pressure of 400 mbar. Then the furnace temperature was set to ramp up to 950°C at 25°C/min and kept for 5 to 30 min under the same flow of mixed gas and vacuum condition. Finally, the system was naturally cooled to room temperature, and a film of light-yellow products on the substrates was prepared.

#### Synthesis of the well-aligned and long ZnO NWs arrays

The ultralong ZnO nanowire arrays were synthesized via a classical CVD process in a single-zone horizontal tube furnace. All chemical reagents were of analytical grade and used without further purification. Firstly, the silicon (100) substrate was washed with absolute alcohol (99.7%) and acetone (99.5%) in an ultrasonic bath, respectively. Then it was etched in a mixed solution of 20 ml ammonia hydroxide (25%), 20 ml H_2_O_2 _(30%), and 100 ml of deionized water at 80°C. An alcoholic solution of zinc acetate dehydrate (0.02 M) was spin-coated on the substrate for five to seven times, followed by annealing in an oven at 120°C for half an hour.

The source material was pure ZnO powder (0.5 g, 99.0%) mixed with graphite (0.5 g, 99.85%). After grinding, the mixtures were spread in a quartz boat placed at the center of the furnace tube (where the inner diameter of the tube is 58 mm). The substrate covered with a film of ZnO seed layer was placed above the source material, and the distance between them was about 2 to 5 mm. Then, 70 sccm of argon gas and 2 sccm of oxygen gas were introduced into the reactor, and the pressure in the tube was adjusted to 400 mbar. The temperature was heated up to 950°C at a rate of 25°C/min and maintained at this temperature for 22 to 60 min. After the reaction, the furnace was cooled naturally to room temperature.

#### Synthesis of the needle-like ZnO NW arrays

The needle-like ZnO nanostructures were prepared by a modified low-temperature solution method. Zinc nitrate hexahydrate (Zn(NO_3_)_2_·6H_2_O, 98%) (1.85 g) and 0.87 g of hexamethylenetetramine (C_6_H_12_N_4_, 98%) were dissolved in 250 mL of deionized water under vigorous magnetic stirring. After a couple of minutes, ethylenediaminetetraacetic acid (EDTA)-2Na (1.16 g) and sodium citrate (0.91 g) were also put into the solution. Finally, the Si substrate coated with nanowire arrays was immersed upside-down in the solution and heated at 80°C for 3 h. After that, the top part of the ZnO nanowire arrays were etched and changed to needle-like structure.

The morphologies of the different ZnO nanostructures were investigated with field emission scanning electron microscopes (FESEM) from JEOL JSM-6700 F (JEOL Ltd., Tokyo, Japan), and the transmission electron microscopy (TEM) images were taken with a JEM 200CX microscope (JEOL Ltd., Tokyo, Japan). The high-resolution TEM (HRTEM) images were taken with a JEOL JEM-2010 F microscope (JEOL, Japan) at an acceleration voltage of 200 kV. The HRTEM specimens were prepared by drop casting the sample dispersion onto a carbon-coated 300-mesh copper grid and were dried under room temperature. X-ray diffraction (XRD) patterns were obtained using a D/MAX-2550 diffractometer (Rigaku, Tokyo, Japan) equipped with a rotating anode and a CuKα radiation source (*λ *= 1.54178 Å). On the other hand, the photoluminescence (PL) measurements were obtained by exciting the samples with a Xe lamp at 325 nm (Hitachi F-7000, Hitachi, Tokyo Japan). Field emission measurement of the as-prepared samples was carried out in a conventional parallel-plate field emission configuration with an anode-to-sample spacing of approximately 150 μm (using glass fibers as spacers for all tests) under a vacuum of 5 × 10^-5 ^Pa.

## Results and discussions

### Morphology of the leaf-like indium-doped ZnO nanostructures

**Figure 1:**
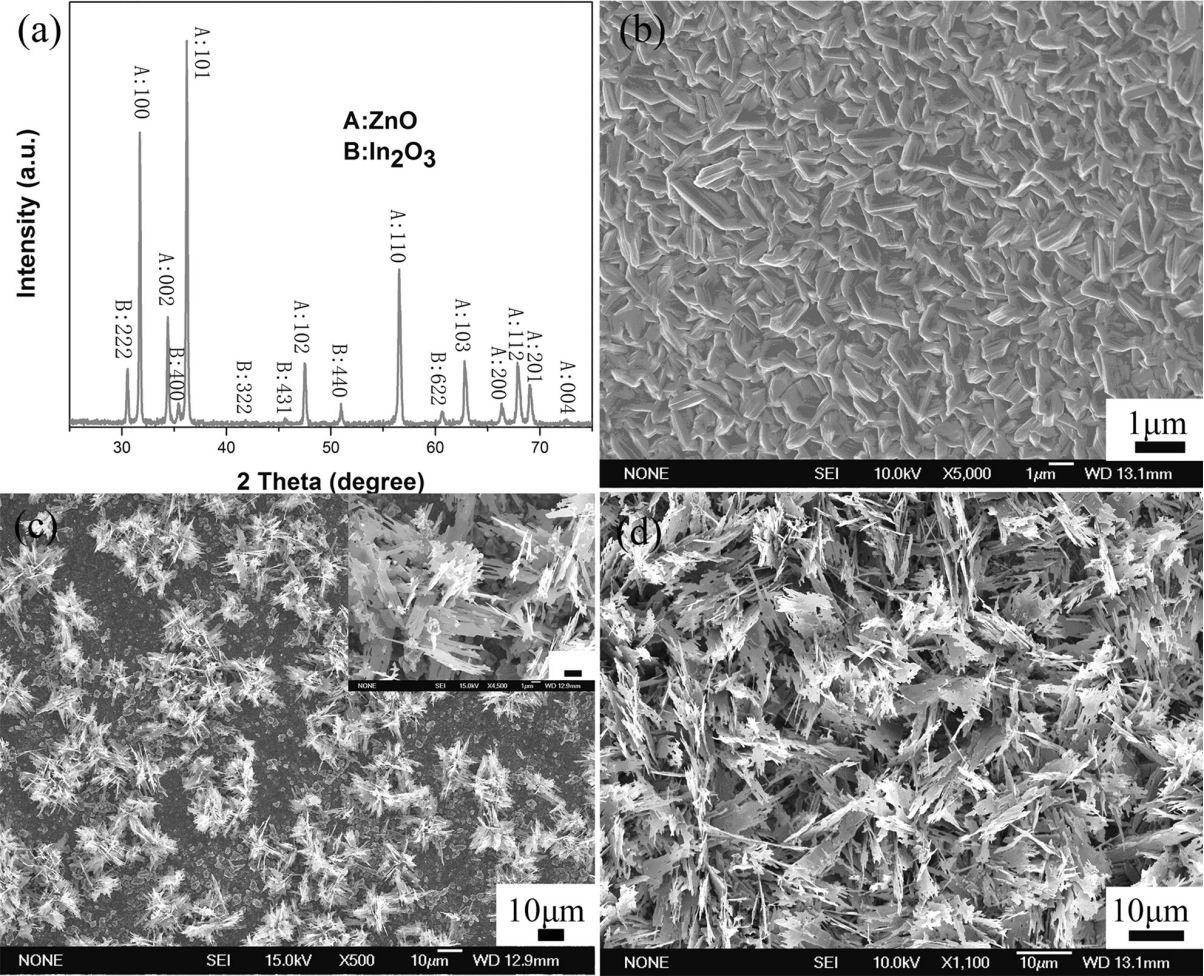
**Morphology of the leaf-like indium-doped ZnO nanostructures**. (**a**) XRD pattern of the leaf-like indium-doped ZnO nanostructures. (**b**), (**c**), and (**d**) SEM images of the leaf-like nanostructures at different reaction times of 5, 10, and 30 min, respectively. The inset in (c) is a zoom of an SEM view of the leaf-like nanostructure, and the scale bar is 1 μm.

The crystal structures of the leaf-like indium-doped ZnO nanostructures were investigated by XRD measurements. As shown in Figure 1a, the main diffraction peaks corresponding to wurtzite ZnO where a = b = 3.24982 Å, c = 5.20661 Å (JCPDS Card No.36-1451) and cubic In_2_O_3 _(JCPDS Card No.06-0416) were observed in the spectrum. Figure 1a also shows that the (101,100) diffraction peaks are much stronger than the other peaks, which indicates that the {101} crystal facets may be the main growth plane of the as-prepared structures. On the other hand, the SEM photographs of the indium-doped ZnO nanostructures in Figure 1b, c, d were prepared with the reaction time of 5, 10, and 30 min, respectively. The figure shows that, with increasing reaction time, the leaf-like structure becomes more apparent where self-assembly flower-like structures composed of nanoparticles and nanobelts were observed on the seed layer as shown in Figure 1c at a longer reaction time of 10 min (initially in Figure 1b, the film consists of microparticles). The width and length of the belt-like structures are around 300 to 600 nm and several micrometers, respectively, as shown in the figure. Finally, after a reaction time of 30 min, large-scale leaf-like structures are self-assembled on the seed layers as shown in Figure 1d where the thickness of the leaf-like branches is about 100 nm.

**Figure 2:**
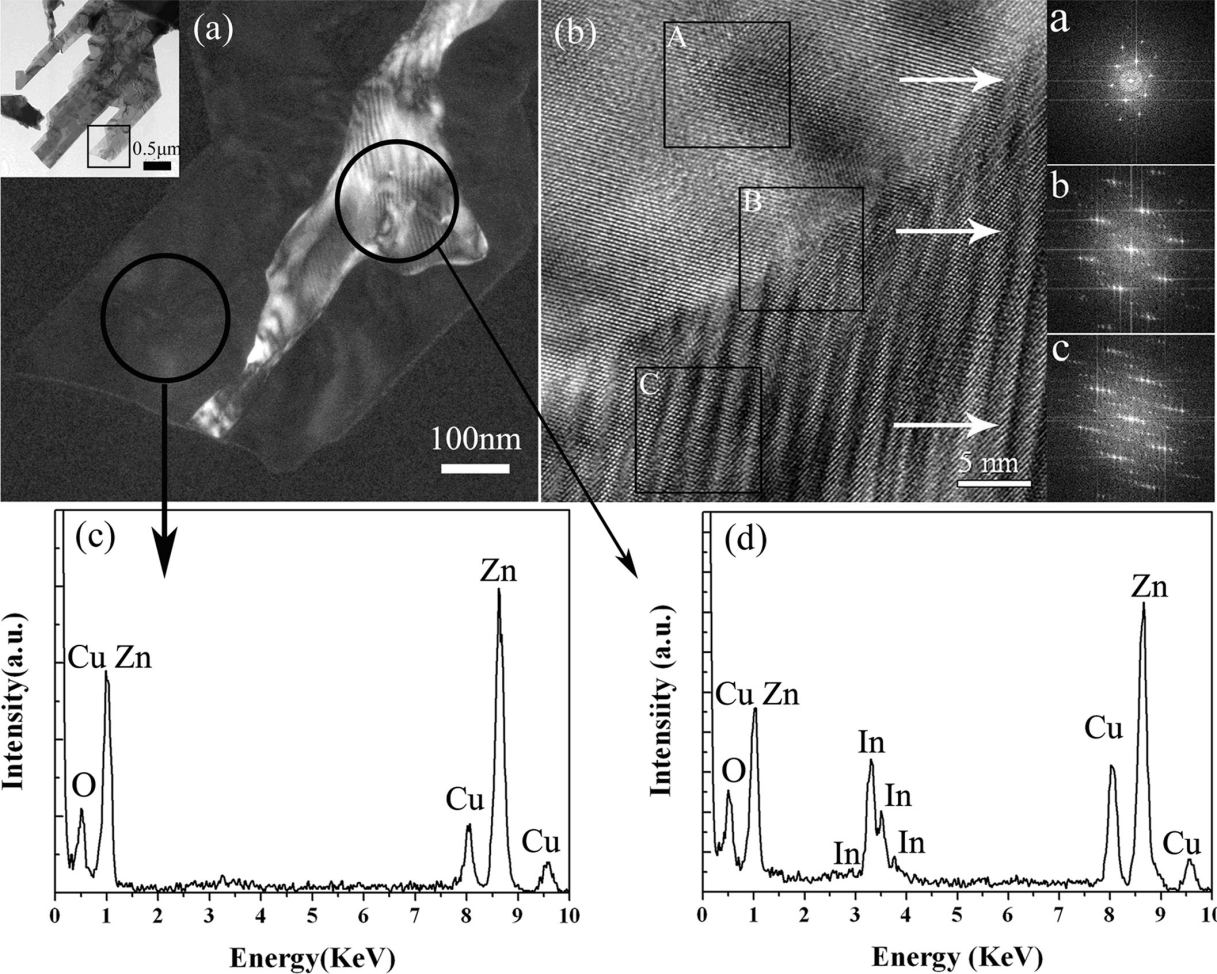
**More detailed measurements of the leaf-like nanostructures**. (**a**) TEM images of the brightfield and darkfield parts of the leaf-like nanostructures, and the inset is the TEM photograph of a small part of the leaf-like sample. (**b**) HRTEM image with which the insets in (b) are the corresponding FFT images taken at the different square areas in the HRTEM image. EDS patterns (**c**) and (**d**) at the two circle regions corresponding to the darkfield and brightfield regions, respectively, in the TEM image.

In order to obtain more detail about the structures and compositions of the leaf-like nanostructures, HRTEM, the fast Fourier transform (FFT), and selected area electron diffraction (SAED) measurements were applied for further characterization of the sample. Figure 2a is the high-resolution TEM image of the area corresponding to the square area as shown in the inset of Figure 2a where the brightfield and the darkfield images correspond to the modulated structures of the In-doped ZnO and single-phase ZnO, respectively. On the other hand, Zn, O, and Cu elements were both observed in the energy dispersive X-ray spectroscopy (EDS) spectrums corresponding to the darkfield (Figure 2c) and brightfield (Figure 2d) parts, respectively. In addition, the element indium was also observed in the brightfield part as shown in Figure 2d. On the other hand, the Cu peak is ascribed to the copper-coated TEM grid, and a close value of molar ratio Zn and In is around 3.5:1 in the brightfield part.

As shown in Figure 2b, HRTEM image taken from the junction between the brightfield and darkfield parts (Figure 2a) indicates different lattice fringes of the modulated and single-phase structures, respectively. The FFT image (Figure 2b) taken from the square marked 'A' from the single lattice fringe shows that it is a typical hexagonal structure consistent with wurtzite ZnO. In addition, the HRTEM image of this structure also shows that the lattice spacing of 0.283 nm between adjacent lattice planes corresponds to the distance between two (10-10) crystal planes. The analysis of the HRTEM image (the square A) agrees with the FFT pattern indicated by 'a'. It is also demonstrated that the top and bottom surfaces of the leaf-like structures are the ± (0001) planes, and the growth orientation is along the a/b axis. On the other hand, the part marked 'B' lies at the junction of the modulated structures and hexagonal ZnO, which corresponds to FFT image 'b', and this part exhibits an approximate hexagonal structure. Conversely, the typically modulated structures taken in square 'C' just accord with the result of the diffraction 'c' spots. It could be possibly due to the reason that the Zn sites in the Zn-O slab are randomly replaced by the In atoms, and hence, local lattice distortions are produced, which induce the periodic diffraction spots. As a result, the brightfield part of the leaf-like nanostructures seems to be In-doped ZnO based. Moreover, the EDS spectrum in Figure 2d corresponding to the brightfield region indicates a higher intensity of the Zn element as compared to the In element, and therefore, this further indicates that the leaf-like nanostructures are In-doped [28]. On the other hand, two sets (or series) of FFT diffraction spots were observed (the first set is the series of slanted bright spots, and the second set is the series of fainter spots between the two slanted rows of bright spots), as shown in the FFT image labeled as 'c' in Figure 2b. These two different kinds of diffraction spots correspond to two parts of the discrepant crystal lattices as observed in the FFT image c in Figure 2b.

**Figure 3:**
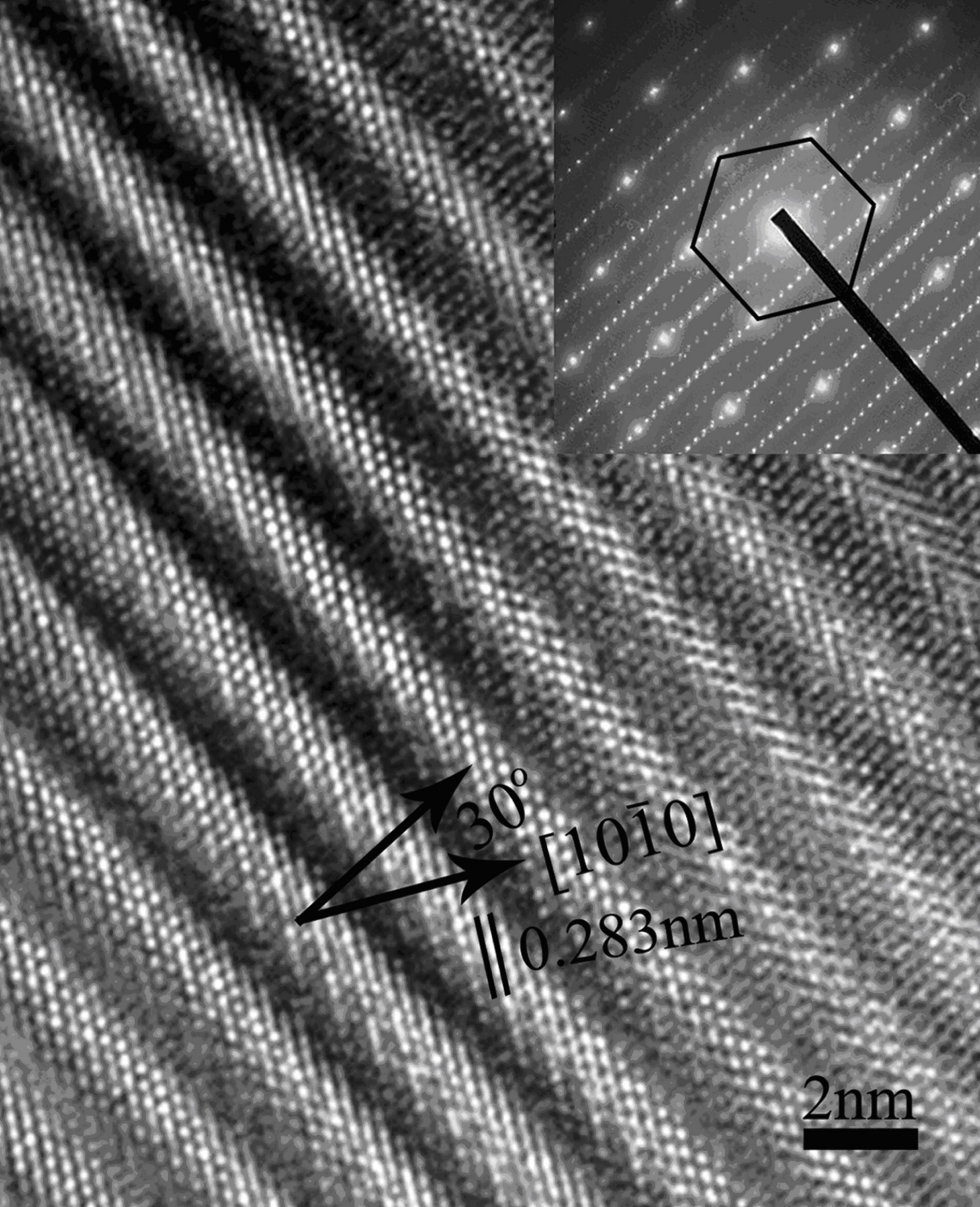
**HRTEM image of the superlattice structure**. The inset of the figure shows the corresponding SAED pattern in the brightfield part of the leaf-like nanostructure.

Figure 3 shows the HRTEM image of the modulated structures in the brightfield part where the wide (bright) and narrow (bright) diagonal lines with a width of 0.283 nm correspond to the In-O layers and In/Zn-O layers, respectively, where the growth direction of the In/Zn-O layers is along the [10-10] direction (crystal ZnO growth direction), as indicated in the figure [14]. However, due to some significant changes to the structures of the leaf-like indium-doped ZnO nanostructures, the stacking layers (bright wide crystal lattice lines) do not grow along the [10-10] orientation but at an angle about 30°, as indicated by the arrow in the HRTEM image and the corresponding SAED pattern (inset of Figure 3). The results are different from previous reports [14]. In addition, Figure 3 illustrates that each wide bright diagonal line corresponds to the In-O layers consist of about seven or eight In/Zn-O layers (width, 0.283 nm). On the other hand, the SAED pattern shows a series of parallel lines and a number of small diffraction spots along the lines, which also indicates the presence of a modulated structure in the brightfield parts of the leaf-like nanostructure. For the In_2_O_3_(ZnO)*_m _*compounds, there is a linear relationship for the width, *d *of the In-O layer (wide bright diagonal line), given by [29]:

(1) d=6.349+2.602mÅ

where *m *is the subscript in the chemical formula of the In_2_O_3_(ZnO)_m _compound.

From the HRTEM image in Figure 3, the average value of *d *is about 19 to 24 Ǻ (width of the bright diagonal line), and hence, the value of *m *as calculated from Equation 1 is between 7 to 8. Thus, the composition of the synthesized leaf-like structures in the brightfield part is estimated as In_2_O_3_(ZnO)_7 _or In_2_O_3_(ZnO)_8_. The results of the EDS spectrum are also applied to detect the component ratios of Zn and In, which show a close value of the molar ratio of Zn:In at approximately 3.5:1 or 4:1. Therefore, the darkfield part of our synthesized leaf-like nanostructure is ZnO, and the brightfield part consists of a modulated structure.

### Growth mechanism of the leaf-like indium-doped ZnO nanostructures

The growth mechanism of nanostructures is usually explained by the vapor-liquid-solid (VLS) and vapor-solid (VS) processes where, according to the VLS growth mechanism, a droplet of the liquid alloy is a key role in reaction process, and hence, the VLS mechanism is also known as catalysis growth. In this work, catalyst was not used in the synthesis of the large-scale ZnO 'leaf' structures by the CVD route; hence, the VS mechanism is appropriate for the explanation of the growth process of the leaf-like indium-doped ZnO structures. In the VS mechanism, the nucleation probability is the critical factor for the formation of the 2-D structures. Based on the reaction condition, the possible factors which could influence the 2-D nucleation process can be expressed as follows:

(2) $$P_{\text{N}}=B\exp\left(-\frac{\pi \sigma^{2}}{K^{2}T^{2}\ln\alpha }\right)$$

where *P*_N _is the nucleation probability, *B *is a constant, *σ *is the surface energy of the solid whisker, *K *is the Boltzmann constant, *T *is the absolute temperature in Kelvin, *α *is the supersaturation ratio determined by *α *= *p*/*p_o _*(and *α *is usually > 1), *p *is the actual vapor pressure, and *p_o _*is the equilibrium vapor pressure corresponding to the temperature T.

According to Equation 2, the factors of *σ*, *α*, and *T *easily give rise to the 2-D nucleation where the higher temperature and larger supersaturation ratios can facilitate the 2-D nucleation, thus further inducing the formation of the sheet-like structure [30]. According to the above-analysis, the Si substrate in the furnace is close to the source materials and lies at a zone where the temperature is high for the CVD process. Therefore, there is a higher distribution of vapor concentration near the substrate. In the reaction process, with increasing reaction temperature, indium and zinc vapor are generated by the carbothermal reduction of In_2_O_3 _and ZnO, respectively. Hence, a small binucleus structure was first synthesized on the substrate by the Zn and In mixture vapor atoms. With the increase of reaction time, newly formed In/Zn atoms continue to deposit on the seed layer and result in the morphologic evolvement from nanoparticles to belt-like and to leaf-like structures on the seed layer, respectively. To keep the system at a lower surface energy, the new arriving In/Zn atoms will react with the pumped-in oxygen gas and are adsorbed on the initial particle-like surfaces. Then, they tend to grow along the longitudinal and transverse growth directions at the same time, which form belt-like structures as well as keep the system at a lower surface energy. At the condition where there is a combination of the high vapor concentration and temperature, the Zn-In diphase will be supersaturated, and thus, the 2-D nucleation gradually forms.

As for the different lengths and widths of the leaf-like branches, it is possible that there is some influence of some thermal or strain instability at the gas-solid interface state which does not involve much energy since the free energy is the same for the equivalent {10-10} planes. In addition, the doped In atoms were obtained by the process of substituting the Zn atoms with the In atoms in the ZnO structures so that the system can be held at a lower energy [31]. By doping ZnO with In, the growth orientations of the resultant nanostructures varied from the highest-energy, low-index planes, and a fast growth along [0001] to the sideway growth [10-10] direction was observed [32,33]. In addition, the new arriving In and Zn atoms in the vapor directly deposit on the surface of In-doped ZnO belt-like structure, and this direct deposition induces the growth of the side faces. Therefore, the seed layer self-assembles on the naked Si substrate and, thus, further induces the formation of leaf-like structures by epitaxial growth.

### PL spectra of the leaf-like indium-doped ZnO nanostructures

**Figure 4:**
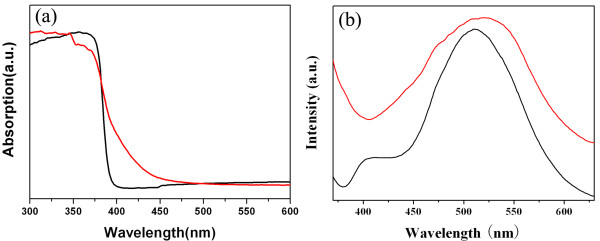
**Room temperature UV and PL spectrum of the (a) leaf-like and (b) wire-like ZnO nanostructures**. The red and black curves correspondingly indicate that the nanostructures are doped with and without indium, respectively.

Figure 4 shows the room temperature PL spectra for leaf-like (red line) indium-doped and wire-like (black line) ZnO nanostructures. According to the theory of semiconductor-metal transition, the bandgap energy *E_g _*decreases when the impurity is more than the Mott critical density [32]; hence, heavy doping leads to an obvious narrowing of *E_g_*. It was observed in Figure 4 that, with In doping, the PL curve becomes broader and the UV-visible band shifts to a longer wavelength, which could be possibly due to the narrowing of *E_g_*. In addition, the doped impurities which induce the increase of the distance between two adjacent In-O layers also result in the further broadening of the full-width at half maximum of the emission peak. A similar result has been reported in In-doped ZnO nanobelts and nanowires [14,34].

### Morphology of the well-aligned and long ZnO NW arrays as well as the needle-like ZnO NW arrays

**Figure 5:**
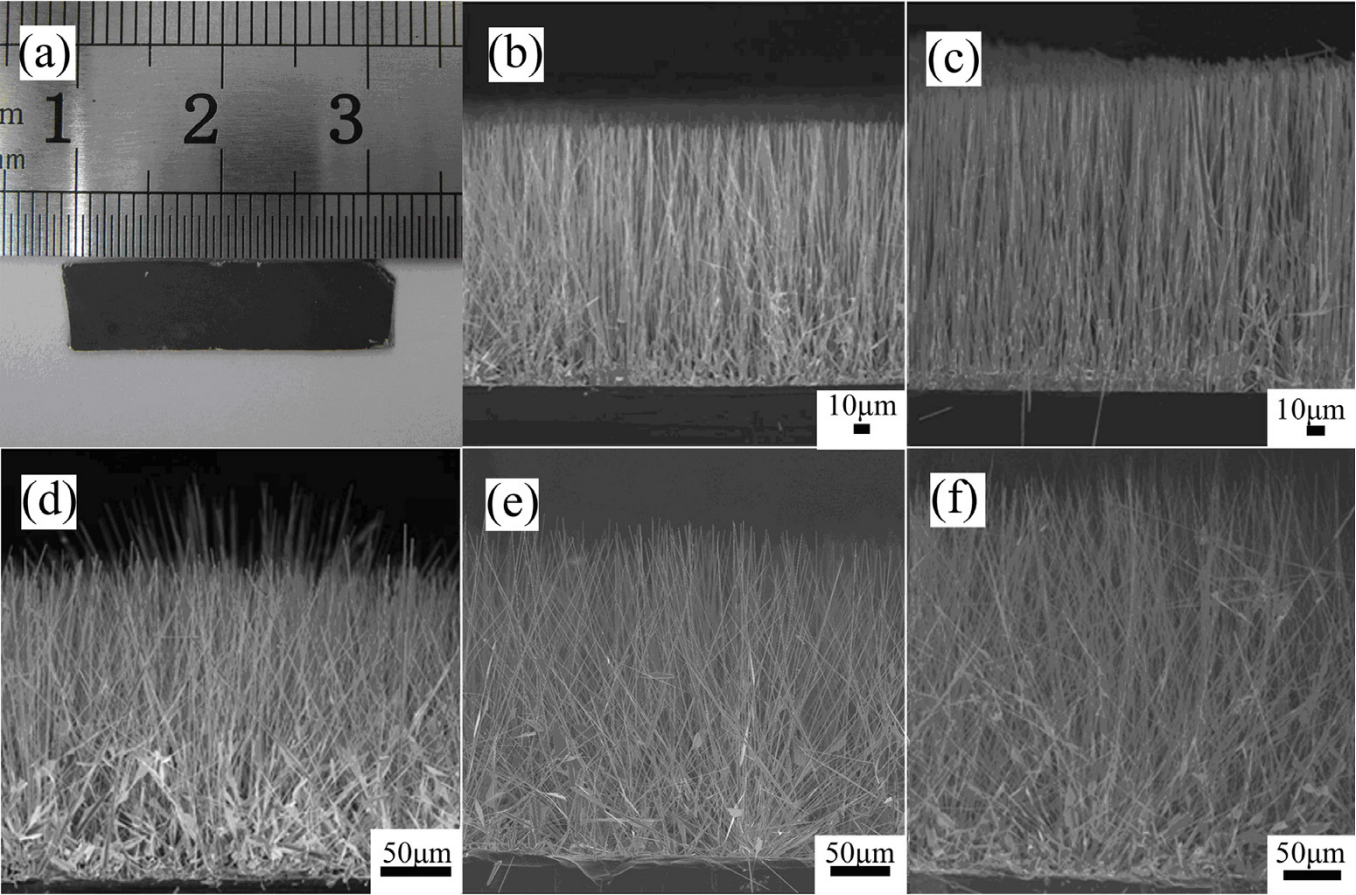
**SEM images of the synthesized arrays of ultralong ZnO nanowires by the CVD process**. The digital image in (**a**) shows a typical sample prepared by the CVD process after a reaction time of 60 min. The SEM images in (**b**), (**c**), (**d**), (**e**), and (**f**) display the length and distribution of the ZnO NW arrays for different reaction times of the CVD process. The lengths of the ZnO NW arrays were about 150, 175, 200, 250, and 310 μm corresponding to the reaction times of 22, 30, 40, 50, and 60 min, respectively.

Figure 5 shows the SEM images of the synthesized arrays of ultralong ZnO nanowires by the CVD process, and the length of the NW array is in the range of 150 to 310 μm and gradually increases with reaction time. From Figure 5b, c, d, e, f, it is seen that, when the length of the ZnO NW arrays increases beyond 175 μm (after a reaction time of 30 min) in Figure 5c, the regularity of the vertical alignment of the NW arrays with respect to the Si substrate becomes poorer.

**Figure 6:**
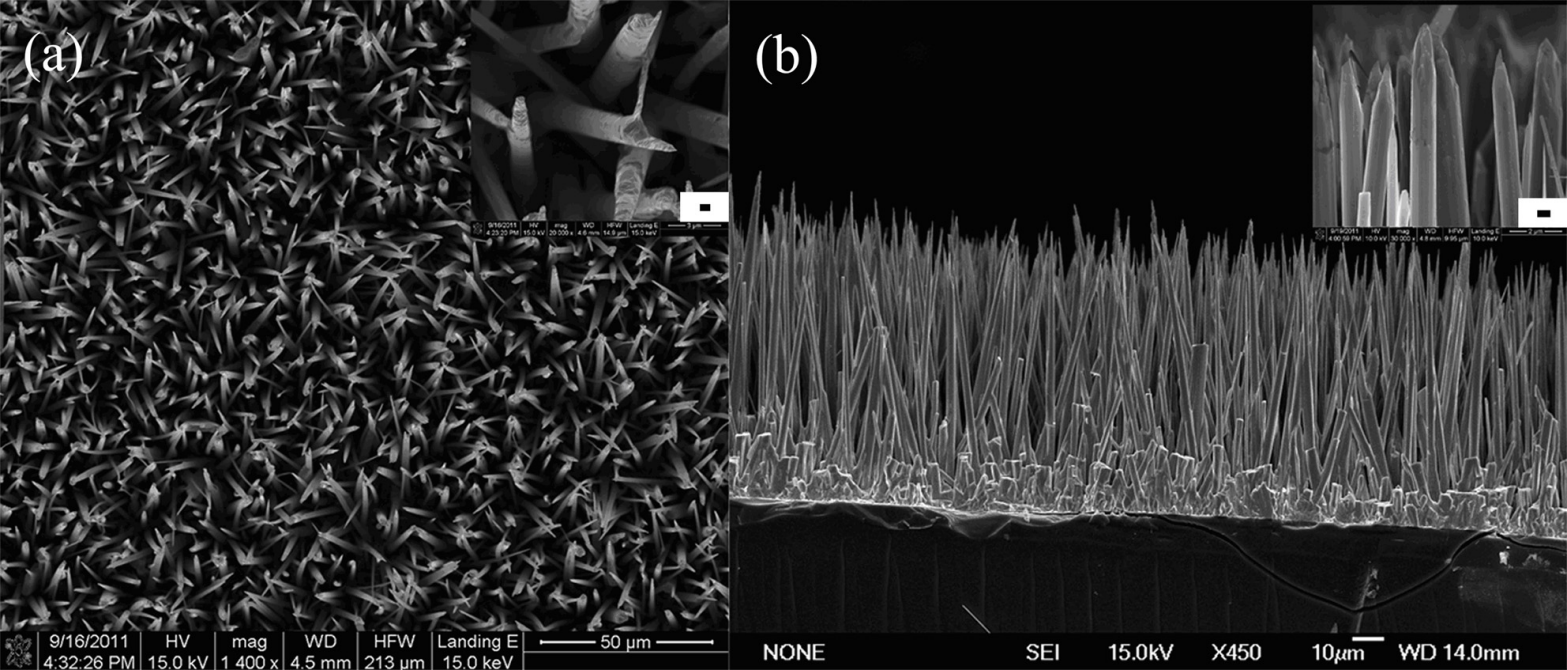
**Typical SEM image of the needle-like ZnO arrays**. (**a**) SEM image of the needle-like ZnO NW arrays; the scale bar in the inset is 500 nm. (**b**) Side-view SEM image of the needle-like ZnO NW arrays; the inset shows the top parts of the NW arrays.

On the other hand, Figure 6 shows the typical SEM image of the needle-like ZnO arrays first prepared by synthesizing arrays of long ZnO NWs followed by chemical etching in the reaction solution. From the figure, the diameter at the top part of the etched NW arrays is about 100 nm. As the needle-like nanowires were obtained by the chemical etching of the nanowire arrays previously prepared by the modified CVD process, hence, the synthesized nanowires are also longer than those prepared by other methods. In addition, the use of this chemical etching route provides a simple, convenient, and low-cost method of obtaining the needle-like nanowires. Therefore, this fabrication technique will offer a method to prepare well-aligned, longer, and sharper nanowire arrays with applications in promising devices.

As shown in Figure 6, ZnO needle-like arrays were prepared by the low-temperature chemical etching route, which shows that surfactants play a key role in the shape evolution of the NWs. In the presence of EDTA-2Na and trisodium citrate, which may preferentially adsorb onto certain surfaces of the ZnO NWs and thus induce the morphology change in the NWs? Hence, we propose that the formation of the needle-like structure may result from the strong chelation reaction of EDTA and citrate ions with positively charged Zn^2+ ^ions in the (0001) plane. Moreover, under the joint action of Zn^2+ ^and surfactants, the concentration of Zn-EDTA and [C_6_H_5_O_7_]_2_Zn_3 _is much more than Zn(OH)_X_, and they are soluble in solution (Equations 3 and 4). Therefore, with the increase of the reaction time, the top part of the ZnO NW arrays will be etched to needle-like structures by the surfactants. With further reaction, as more of the complexes begin to form and dissolve into the reaction solution, the top part of the ZnO NW arrays were modified to be tapering in shape.

(3) $$\text{EDTA}-\text{2Na}+\text{Z}{\text{n}}^{2+}↔\text{Zn}-\text{EDTA}+\text{2N}{\text{a}}^{+}$$

(4) $$\text{2}\left[{\text{C}}_{\text{6}}{\text{H}}_{\text{5}}{\text{O}}_{\text{7}}\right]-\text{3Na}\;+\;\text{3Z}{\text{n}}^{\text{2}+}\;↔\;{\left[{\text{C}}_{\text{6}}{\text{H}}_{\text{5}}{\text{O}}_{\text{7}}\right]}_{\text{2}}\text{Z}{\text{n}}_{\text{3}}\;+\;\text{6N}{\text{a}}^{+}$$

### Field emission properties of the different ZnO nanostructures

For an analysis of the field emission properties of the different ZnO nanostructures, the following Fowler-Nordheim (F-N) equation would have to be used:

(5) $$J=\frac{A\beta^{2}E^{2}}{\varphi }\exp\left(-\frac{B\varphi^{3/2}}{\beta E}\right)$$

where *J *is the current density, *E *is the applied field strength, *A *and *B *are constants with the values of 1.56 × 10^-10 ^AV^-2 ^eV and 6.83 × 10^3 ^V(eV)^-3/2 ^μm^-1^, respectively, *ϕ *is the work function of the emitter which was taken as 5.4 eV for ZnO from the literature [35], and *β *is the so-called field-enhancement factor, which reflects the ability of the emitters to enhance the local electric field. The field-enhanced factors *β *can be calculated from the formula *β *= -*Bϕ*^3/2^/*S*, where *S *is the slope of the F-N plot.

**Figure 7:**
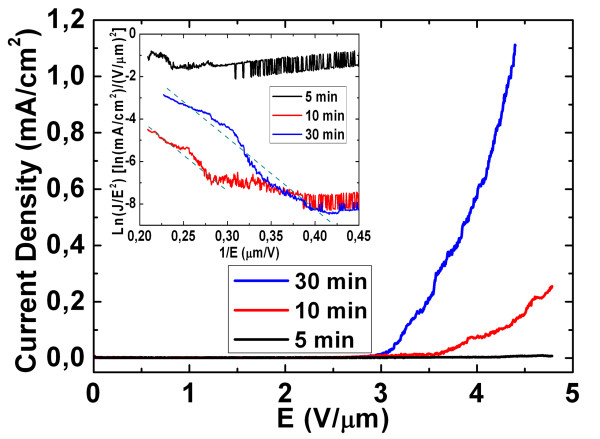
***J*-*E *curves of the leaf-like ZnO nanostructures at reaction times 5, 10, and 30 min**. The inset in the figure shows the corresponding Fowler-Nordheim plots.

Figure 7 shows the typical leaf-like ZnO nanostructure field emission current density versus the applied field (*J*-*E*) curves and the corresponding F-N plots (the inset of Figure 7) at different reaction time, respectively. In Figure 7, when the reaction time increases from 5 to 30 min, the turn-on field (defined as the applied field at the emission current density of 10 μA/cm^2^) is decreased to 2.94 V/μm (which is lower than the previous reports [32-34]). On the other hand, the emission current density of the curve corresponding to the reaction time of 30 min is about 1 mA/cm^2 ^at an applied field of about 4.35 V/μm (so-called threshold field); thus, the 30-min sample possesses better field emission properties as compared to the 5- and 10-min samples. One possible reason could be that the 30-min leaf-like ZnO nanostructure is comprise of more rod-shaped structures (or tips) as compared to the other two samples. On the other hand, the increased indium doping in the 30-min sample as compared to others could have led to further bandgap narrowing and the moving closer of the Fermi level towards the bottom of the conduction band. Hence, the increased carrier concentration in the 30-min leaf-like ZnO nanostructure could have led to the increase of the field emission current and better field emission properties as observed in Figure 7.

Based on Equation 5, the experimental *β *value for both the 30- and 10-min leaf-like ZnO nanostructure samples are close to 2,800. Conversely for the 5-min sample, the corresponding *J*-*E *curve in Figure 7 is observed to be nonlinear, and it could be possibly due to the reason that the sample consists of microparticles, thus leading to a relatively high turn-on field and a nonlinear relationship.

**Figure 8:**
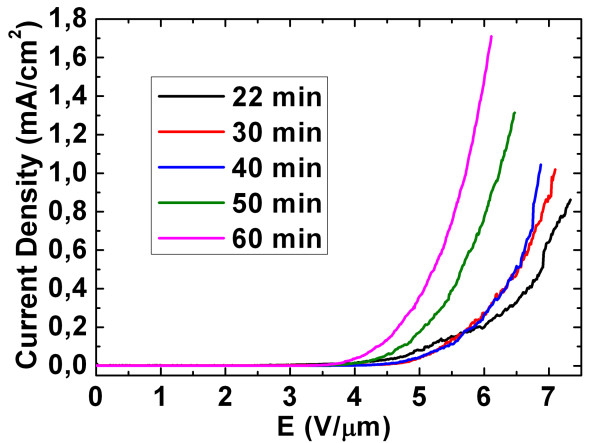
***J*-*E *curves of the long ZnO nanowire arrays at different reaction times**.

**Figure 9:**
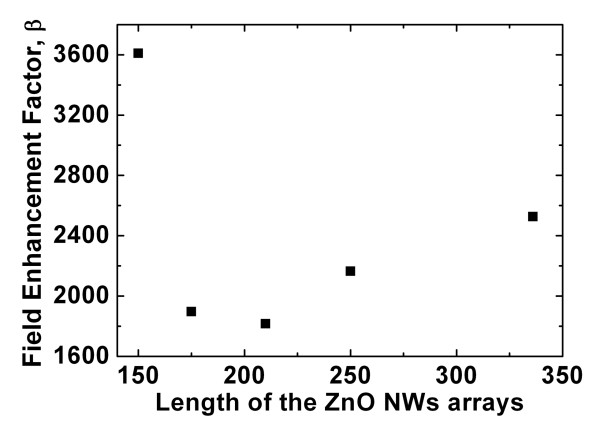
**Field-enhanced factors of the long ZnO NW arrays of different length**.

On the other hand, Figures 8 and 9 show the plot of the emission current density versus the electric field of the long ZnO nanowire arrays at different reaction time (during the CVD process) and their corresponding field-enhancement factors, respectively. In Figure 8, when the reaction time increases from 22 to 60 min, the turn-on field (defined as the applied field at the emission current density of 10 μA/cm^2^) decreases from 4.56 to 3.69 V/μm, respectively. In addition, the emission current density of the curves also decreases from 7.09 to 5.72 μA/cm^2 ^at the threshold field. Hence, the 60-min sample possesses better field emission properties as compared to the others (22- to 50-min samples). Based on the formula in Equation 5, the experimental *β *values for the 22- to 60-min samples are plotted in Figure 9. Comparing the experimental *β *value between the indium-doped leaf-like ZnO nanostructures and the long ZnO NW arrays, it was seen that the 30-min indium-doped leaf-like nanostructures possess a slightly higher *β *value as compared to most of the long ZnO NW array samples (except for the 22-min sample whose average length of the NWs is about 150 μm in Figure 9).

It is well-known that the field emission properties of the 1-D nanostructures depend on the tip morphology, density, and the aspect ratio of the length to diameter (*h*/*d*) corresponding to the NWs [36]. Although the long ZnO NW arrays have a relatively bigger diameter, their higher aspect ratios, however, would help to improve the field-enhancement factors. Nonlinearity is also observed in the F-N plots corresponding to the 22- to 60-min samples, which could be possibly due to the high density of the NW arrays in the samples.

The turning point in the plot of the field-enhancement factors versus the length of ZnO NWs can be explained as follows. To the first approximation, the field-enhancement factor, *β *can be given by *β *= *h*/*r *[36] where *h *is the length or height and *r *is the radius of the NWs, respectively. As a result, as the length of the NWs increases, this will increase the value of *β*. On the other hand, it was also experimentally observed (by Wong et al.) that the concentration of the oxygen vacancies *V_o _*is positively correlated with the increase in the length of the ZnO NWs [37]. This increase of the concentration of *V_o _*corresponding to the longer ZnO NWs [37] will then lead to an increased barrier for electron emission [38], thus leading to a decrease of the corresponding experimentally observed *β *value for the longer ZnO NWs. Therefore, these two contrasting mechanism which result in the increase and decrease of the value of *β*, respectively, will lead to a turning point in Figure 9 as the length of the ZnO NWs varies.

**Figure 10:**
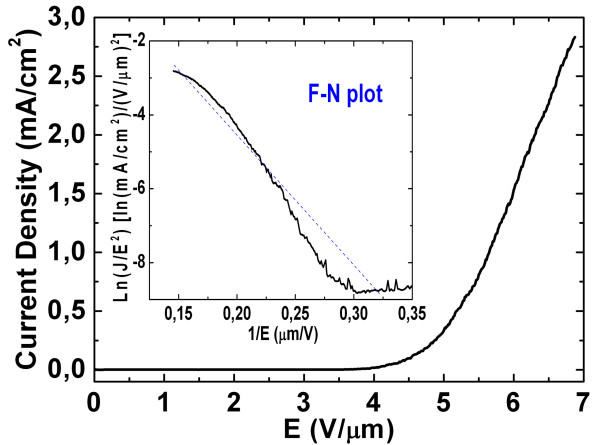
**Field emission current density as a function of the applied field for the needle-like sample**. The inset in the figure shows the corresponding F-N plot.

Figure 10 shows the current density-electric field (*J*-*E*) plot corresponding to the needle-like ZnO arrays. The turn-on electric field of the ZnO needle-like arrays is about 3.87 V/μm at a current density of 10 μA/cm^2^, while the electric field corresponding to the current density of 1 mA/cm^2 ^is 5.65 V/μm (the *β *value for the needle-like arrays is about 2,284), and these results are close to the 60-min ZnO NW arrays sample. However, although the smaller diameter at the top part of the needle-like structures could easily induce much more electron emission, the needle-like ZnO NW arrays are densely packed. Hence, the screening effect between the neighboring emitters would decrease the experimentally observed value of *β *corresponding to the needle-like ZnO NW arrays [36] as compared to the long ZnO NW arrays. Table 1 summarizes the field emission properties of the three different synthesized ZnO nanostructures, and it shows that the leaf-like ZnO nanostructures is most suitable for field emission due to its lowest turn-on and threshold field as well as its relatively high field-enhancement factor. Hence, the synthesized leaf-like structure will be promising in the applications of electron sources or flat panel displays.

**Table 1:** Comparison of the field emission properties between the different ZnO nanostructures

Type of ZnO nanostructure	Turn-on field (V μm^-1^)	Threshold field (V μm^-1^)	*β*
Leaf-like (30 min)	2.94	4.35	2,800
Long NWs arrays (60 min)	3.69	5.72	2,526
Needle-like	3.87	5.65	2,284

## Conclusion

The indium-doped leaf-like ZnO nanostructures, the well-aligned long ZnO NW arrays, and the needle-like arrays were synthesized on the Si substrates. The near distance between the source and the Si substrate during the CVD process gives rise to a larger supersaturation ratio for the easier nucleation of the ZnO and In atoms. This will help the formation of the indium-doped ZnO leaf-like nanostructure. On the other hand, the morphology characterization shows that the indium-doped ZnO leaf-like nanostructure consists of a brightfield and darkfield region. The brightfield and darkfield regions correspond to the modulated and single-phase structure, respectively, in the indium-doped ZnO leaf-like nanostructure. Owing to indium doping, the emission peak of the photoluminescence spectra corresponding to the leaf-like structures was observed to be broadened and shifted to higher wavelengths. On the other hand, with the use of the CVD process, well-aligned and long ZnO NW arrays can be obtained. Subsequently, with chemical etching, needle-like NW arrays can be obtained, which is a relatively low-cost and convenient method.

The field emission properties of the different nanostructures were also investigated, and it was observed that the field-enhancement factor varies between the different ZnO nanostructures. From the field emission properties corresponding to the different nanostructures, the indium-doped leaf-like nanostructures are suitable for use as a field emitter in microelectronic devices. This is due to its lowest turn-on and threshold field as well as its relatively high field-enhancement factor among the different synthesized nanostructures. On the other hand, the variation of the field-enhancement factor for the long ZnO NW arrays with different lengths is related to the variation in the concentration of oxygen vacancies with the length of the NW arrays.

## Competing interests

The authors declare that they have no competing interests.

## Authors' contributions

KMW and YF contributed equally in conceiving of the study and its design and coordination, in conducting all the experiments (synthesizing the nanostructures), measurements, results interpretation, and drafting of the manuscript. YL supervised all of the study. All authors read and approved the final manuscript.
